# A practical scoring model to predict the occurrence of critical illness in hospitalized patients with SARS-CoV-2 omicron infection

**DOI:** 10.3389/fmicb.2022.1031231

**Published:** 2022-12-19

**Authors:** Yao Zhang, Jiajia Han, Feng Sun, Yue Guo, Yifei Guo, Haoxiang Zhu, Feng Long, Zhijie Xia, Shanlin Mao, Hui Zhao, Zi Ge, Jie Yu, Yongmei Zhang, Lunxiu Qin, Ke Ma, Richeng Mao, Jiming Zhang

**Affiliations:** ^1^Department of Infectious Diseases, Shanghai Key Laboratory of Infectious Diseases and Biosafety Emergency Response, National Medical Center for Infectious Diseases, Huashan Hospital, Fudan University, Shanghai, China; ^2^Department of Respiratory Medicine, Huashan Hospital North, Fudan University, Shanghai, China; ^3^Department of Emergency and Acute Critical Care, Huashan Hospital North, Fudan University, Shanghai, China; ^4^Department of General Surgery, Huashan Hospital, Cancer Metastasis Institute, Fudan University, Shanghai, China; ^5^Shanghai Institute of Infectious Diseases and Biosecurity, Key Laboratory of Medical Molecular Virology (MOE/MOH), Shanghai Medical College, Fudan University, Shanghai, China; ^6^Department of Infectious Diseases, Jing’An Branch of Huashan Hospital, Fudan University, Shanghai, China

**Keywords:** COVID-19, severe acute respiratory syndrome coronavirus 2, omicron, critical illness, risk factors

## Abstract

**Background:**

The variants of severe acute respiratory syndrome coronavirus 2 (SARS-CoV-2) have emerged repeatedly, especially the Omicron strain which is extremely infectious, so early identification of patients who may develop critical illness will aid in delivering proper treatment and optimizing use of resources. We aimed to develop and validate a practical scoring model at hospital admission for predicting which patients with Omicron infection will develop critical illness.

**Methods:**

A total of 2,459 patients with Omicron infection were enrolled in this retrospective study. Univariate and multivariate logistic regression analysis were performed to evaluate predictors associated with critical illness. Moreover, the area under the receiver operating characteristic curve (AUROC), continuous net reclassification improvement, and integrated discrimination index were assessed.

**Results:**

The derivation cohort included 1721 patients and the validation cohort included 738 patients. A total of 98 patients developed critical illness. Thirteen variables were independent predictive factors and were included in the risk score: age > 65, C-reactive protein > 10 mg/L, lactate dehydrogenase > 250 U/L, lymphocyte < 0.8*10^^9^/L, white blood cell > 10*10^^9^/L, Oxygen saturation < 90%, malignancy, chronic kidney disease, chronic cardiac disease, chronic obstructive pulmonary disease, diabetes, cerebrovascular disease, and non-vaccination. AUROC in the derivation cohort and validation cohort were 0.926 (95% CI, 0.903–0.948) and 0.907 (95% CI, 0.860-0.955), respectively. Moreover, the critical illness risk scoring model had the highest AUROC compared with CURB-65, sequential organ failure assessment (SOFA) and 4C mortality scores, and always obtained more net benefit.

**Conclusion:**

The risk scoring model based on the characteristics of patients at the time of admission to the hospital may help medical practitioners to identify critically ill patients and take prompt measures.

## Introduction

Although two years have passed since the coronavirus disease 2019 (COVID-19) caused by severe acute respiratory syndrome coronavirus 2 (SARS-CoV-2) outbroke, various variants have been identified in different parts of the world (Zhu et al., 2020; Islam et al., 2021). The Omicron variant, in particular, rapidly gained dominance through its ability to fast spread (Petersen et al., 2022). The cumulative diagnosed cases have reached over 600,000 and close to 600 cases have died with or from COVID-19 as of 30 June 2022 since the recent Omicron outbreak in Shanghai (Ye et al., 2022; Yuan et al., 2022). The treatment of patients critically ill with SARS-CoV-2 infection is one of the main challenges facing clinicians and there is a need to adopt reliable predictors of the severity of patients to identify and treat the most severe patients in the early stage.

Previous studies have described the clinical characteristics of SARS-CoV-2 infection and explored the associations of epidemiological, comorbidity factors, imaging with severity and prognosis of COVID-19 (Chen et al., 2020; Huang C. et al., 2020; Liang et al., 2020a; Lacap et al., 2022). A study with 1,590 patients revealed that comorbidities were associated with poorer clinical outcomes (Guan et al., 2020). Older age, male sex, and active cancer also contributed to the increased mortality in a prospective, observational, single-center study (Başaran et al., 2022). Moreover, various studies have gathered evidence on the levels of serum cytokines, and the effect of concentrations of trace elements (Han et al., 2020; Heller et al., 2021). However, studies of COVID-19 prognostic factors have focused on radiological examinations obtained following admission or immunological indicators and other expensive examination indicators. Tools that provide practical, accurate, and low-cost risk estimates are needed, as estimates requiring extensive testing or imaging increase the burden on healthcare systems already operating at capacity. Previous published models tend not to include clinical variables obtained from history and examination carried out on initial assessment, as well as vaccination status. Since many variables of the pandemic have changed, widespread vaccination uptake has affected the incidence of severe COVID-19, and emerging variants of concern also have differing spectra of severity. Predicting the risk of critical illness with COVID-19 could help to identify those patients who require the most urgent help, and it may be of great utility for healthcare professionals to efficiently perform triage of patients, personalize treatment, and monitor clinical progress.

A large-scale outbreak poses significant challenges to the healthcare system, and the ability to identify patients who are most at risk of developing severe disease upon admission is critical for effective triage, management, and discharge decision making. In this study, we aimed to elucidate potential risk factors based on a cohort of Chinese patients with SARS-CoV-2 infection to attempt to identify patients at the time of hospital admission who are probably to develop critical illness. In addition, the accuracy of the novel risk score was compared with CURB-65, sequential organ failure assessment (SOFA), and 4C mortality scores.

## Patients and methods

### Patients

**FIGURE 1:**
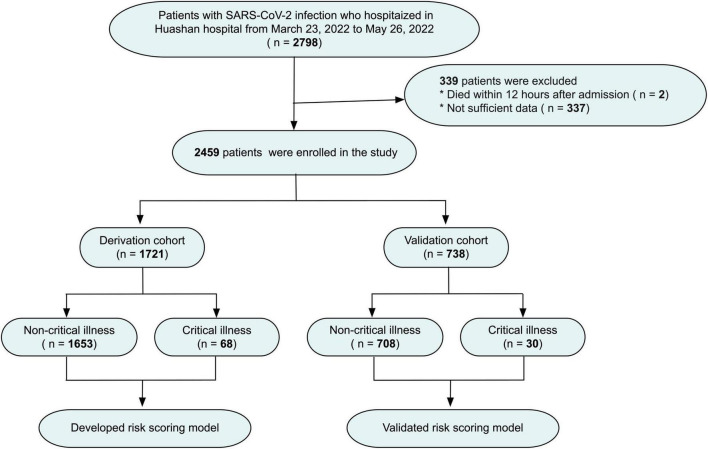
Flow chart of the study design.

Between March 23, 2022 to May 26, 2022, 2459 of the 2798 confirmed SARS-CoV-2 Omicron infection patients, who hospitalized in Huashan Hospital of Fudan University were enrolled in the study. SARS-COV-2 infection was confirmed by positive real-time reverse-transcription polymerase-chain-reaction (PCR) assay for nasal and pharyngeal swab specimens. Patients died within 12 h after admission and without sufficient data were excluded. Critical cases were defined according to the World Health Organization as acute respiratory distress syndrome (ARDS), sepsis, septic shock, or other conditions that would normally require the provision of life-sustaining therapies such as mechanical ventilation or vasopressor therapy. The entire cohort was divided into the derivation (*n* = 1721) cohort and the validation (*n* = 738) cohort chronologically (Figure 1).

This study was approved by the ethics committee of Huashan Hospital of Fudan University. Written informed consent was waived in consideration of the retrospective nature of the study. The clinical diagnosis and treatment complied with the Helsinki declaration.

### Data collection

The study data were obtained from electronic medical records and they included demographics (age and gender), vital signs, clinical symptoms on admission, vaccination history, comorbidities, laboratory findings on admission (routine blood analyses, biochemical markers, coagulative function and so on), oxygen support need (high flow oxygen, non-invasive and invasive ventilation), and medications (ritonavir/darunavir, glucocorticoid, heparin, and vasoactive drugs). Chronic cardiac disease is summarized as established diagnoses of coronary artery disease, previous episodes of myocardial infarction and chronic heart failure. Chronic kidney disease was defined as repeatedly measured glomerular filtration rate (GFR) < 60 mL/min prior to present hospitalization.

### Statistical analysis

All statistical analyses were performed using SPSS software for Windows (version 26.0; IBM, Endicott, NY, USA). The continuous variables in normal distribution were presented as means ± standard deviations (SDs), and median (interquartile ranges) were used for data that are not normally distributed. The categorical variables were summarized as a proportion (%). Student’s *t*-test, Wilcoxon’s non-parametric test, and the χ2-test or Fisher’s exact test were applied to normally distributed data, non-normal distribution continuous variables and the categorical variables, respectively. Univariable and multivariable logistic regression analyses were conducted to calculate the parameters predicting the occurrence of critical illness. All variables with *p* < 0.05 in the univariate analysis were selected for further multivariate analysis to identify independent predictors. Areas under the receiver operating characteristic curve (ROC) were used to estimate the predictive accuracy, and compared using Delong test. Continuous net reclassification improvement (NRI) and integrated discrimination improvement (IDI) were calculated using the package “PredictABEL” in the R statistical computing language (version 3.6.3; R Foundation for Statistical Computing; Auckland University, Auckland, New Zealand). Two-tailed *p* value < 0.05 was considered statistically significant.

## Results

### Patient characteristics

A total of 2459 patients (out of 2,798 screened) were suitable for inclusion in this study. 29 of 2459 patients had sepsis and/or ARDS on admission. A total of 98 patients eventually developed critical illness and 2,361 did not. The median time from hospitalization to develop critical illness was 6 days. The characteristics of these two groups including age, blood test results, comorbidities, clinical symptom, and COVID-19 vaccination status on admission were shown in Supplementary Figure 1.

A total of 1,721 patients who hospitalized before May 1 constituted the derivation cohort. In the derivation cohort, 23 of 1,721 patients were considered to be severe on admission and a total of 68 patients eventually developed critical illness. A total of 20 patients died during hospitalization. Demographic and clinical characteristics of the derivation cohort were available in Table 1. The age (81 vs. 64, *p* < 0.001) and proportion of male (63.2% vs. 47.5%, *p* = 0.011) in critical patients were significantly higher than those in non-critical patients. Critical patients had higher alanine aminotransferase (ALT), aspartate transaminase (AST), total bilirubin (TB), white blood cell (WBC), creatinine, lactate dehydrogenase (LDH), D-dimer, C-reactive protein (CRP), procalcitonin (PCT), Cardiac troponin T (cTnT), and N-terminal forebrain natriuretic peptide (NT-proBNP), but significantly lower lymphocyte count and estimated glomerular filtration rate (eGFR) compared with non-critical patients. Hypertension was the most common comorbidity, followed by diabetes and chronic cardiac disease. Compared with non-critical patients, comorbidities and clinical symptoms (fever, cough, expectoration, pharyngalgia and feeble) were more common in critical patients. There were more patients unvaccinated in critical group than non-critical group (80.9% vs. 38.1%, *p* < 0.001). In addition, among patients who did develop critical illness, the proportion of using heparin, glucocorticoid, and vasoactive drugs was significantly higher than the patients who did not develop critical illness.

**TABLE 1:** Demographics and clinical characteristics of patients in the derivation cohort.

	Total	Non-critical illness	Critical illness	*p* value
Total (*n*)	1721	1,653	68	
Male	829 (48.2%)	786 (47.5%)	43 (63.2%)	0.011
Age (years)	64.0 (49.0-73.0)	64.0 (48.0-73.0)	81.0 (71.0-90.0)	<0.001
**Laboratory findings on admission**				
ALT (U/L)	16.0 (11.0-25.0)	16.0 (11.0-24.0)	19.5 (11.0-36.5)	<0.001
AST (U/L)	20.0 (16.0-26.0)	20.0 (15.0-26.0)	28.0 (19.0-48.5)	<0.001
TB(umol/L)	8.0 (5.6-11.4)	7.9 (5.6-11.3)	9.9 (7.1-14.7)	<0.001
WBC (*10^^9^/L)	5.2 (3.9-6.8)	5.1 (3.9-6.7)	7.5 (5.6-11.7)	<0.001
Lymphocyte (*10^^9^/L)	1.2 (0.8-1.7)	1.3 (0.9-1.7)	0.6 (0.5-1.0)	<0.001
Creatinine (umol/L)	76.0 (61.0-103.0)	75.0 (60.0-99.0)	126.5 (70.8-346.0)	0.003
eGFR (mL/min)	84.1 (57.8-104.1)	84.9 (61.9-104.5)	44.6 (14.2-86.1)	<0.001
LDH (U/L)	195.0 (166.0-232.5)	193.0 (165.0-229.0)	294.0 (219.5-398.0)	<0.001
D-dimer (FEUmg/L)	0.6 (0.3-1.1)	0.6 (0.3-1.0)	2.4 (1.1-4.6)	<0.001
CRP (mg/L)	8.0 (5.0-26.1)	6.9 (5.0-21.9)	72.4 (35.6-111.0)	<0.001
PCT (ng/ml)	0.1 (0.1-0.5)	0.1 (0.1-0.4)	0.6 (0.2-2.8)	<0.001
cTnT (ng/ml)	0.010 (0.007-0.033)	0.012 (0.007-0.028)	0.079 (0.035-0.179)	<0.001
NT-proBNP (pg/ml)	137.0 (47.5-877.5)	124.0 (45.1-599.0)	2724.5 (1052.5-19420.3)	<0.001
**Comorbidities, *N* (%)**				
Hypertension	805 (46.8%)	762 (46.1%)	43 (63.2%)	0.006
Diabetes	433 (25.2%)	401 (24.3%)	32 (47.1%)	<0.001
Chronic cardiac disease	276 (16.0%)	247 (14.9%)	29 (42.6%)	<0.001
Cerebrovascular disease	201 (11.7%)	182 (11.0%)	19 (27.9%)	<0.001
Chronic kidney disease	260 (15.1%)	234 (14.2%)	26 (38.2%)	<0.001
COPD	66 (3.8%)	60 (3.6%)	6 (8.8%)	0.029
Liver disease	44 (2.6%)	39 (2.4%)	5 (7.4%)	0.011
Malignancy	131 (7.6%)	119 (7.2%)	12 (17.6%)	0.001
**Symptoms, *N* (%)**				
Fever	367 (21.3%)	342 (20.7%)	25 (36.8%)	0.002
Cough and expectoration	503 (29.2%)	470 (28.4%)	33 (48.5%)	< 0.001
Pharyngalgia	54 (3.1%)	48 (2.9%)	6 (8.8%)	0.006
Feeble	210 (12.2%)	196 (11.9%)	14 (20.6%)	0.031
Runny nose	33 (1.9%)	30 (1.8%)	3 (4.4%)	0.126
**Vaccination status, *N* (%)**				
Unvaccinated	685 (39.8%)	630 (38.1%)	55 (80.9%)	< 0.001
Partially vaccinated	53 (3.1%)	52 (3.1%)	1 (1.5%)	0.433
Fully vaccinated	455 (26.4%)	452 (27.3%)	3 (4.4%)	0.001
Booster vaccination	528 (30.7%)	519 (31.4%)	9 (13.2%)	0.001
**Oxygen support need, *N* (%)**				
Non-invasive ventilation	333 (19.3%)	307 (18.6%)	26 (38.2%)	<0.001
High flow oxygen	35 (2.0%)	20 (1.2%)	15 (22.1%)	<0.001
Mechanical ventilation	28 (1.6%)	0 (0%)	28 (41.2%)	<0.001
**Medications, *N* (%)**				
Ritonavir/Darunavir	620 (36.0%)	591 (35.8%)	29 (42.6%)	0.246
Heparin	419 (24.3%)	381 (23.0%)	38 (55.9%)	<0.001
Glucocorticoid	100 (5.8%)	75 (4.5%)	25 (36.8%)	<0.001
Vasoactive drugs	38 (2.2%)	0 (0%)	38 (55.9%)	<0.001

Values are expressed as median (IQR) or number of patients (%). ALT, alanine aminotransferase; AST, aspartate transaminase; TB, total bilirubin; WBC, white blood cell; eGFR, estimated glomerular filtration rate; LDH, lactate dehydrogenase; CRP, C-reactive protein; PCT, procalcitonin; cTnT, cardiac troponin T; NT-proBNP, N-terminal forebrain natriuretic peptide; COPD, chronic obstructive pulmonary disease.

### Predictors of critical illness risk in patients with omicron infection

The preliminary univariate and multivariate logistic regression analyses were used to identify predictors of critical illness with laboratory parameters and oxygen saturation at admission. The results of the univariate analyses showed that ALT, AST, TB, WBC, lymphocyte, creatinine, LDH, D-dimer, CRP, PCT, cTnT, NT-proBNP, and oxygen saturation were associated with critical illness. Multivariate analyses indicated that the WBC count (odds ration [OR]: 1.149; 95% confidence interval [CI]: 1.046-1.261; *p* = 0.004), lymphocyte count (OR: 0.297; 95% CI: 0.139-0.637; *p* = 0.002), LDH (OR: 1.008; 95% CI: 1.004-1.011; *p* < 0.001), CRP (OR: 1.007; 95% CI: 1.001-1.014; *p* = 0.020), and oxygen saturation on room air (OR: 0.955; 95% CI: 0.922-0.990; *p* = 0.011) were significant independent predictors of critical illness (Table 2).

**TABLE 2:** Univariate and multivariate analysis of laboratory parameters and oxygen saturation at admission predicting critical illness.

Predictors	Univariate analysis		Multivariate analysis	
	**OR (95% CI)**	***p*** ** value**	**OR (95% CI)**	***p*** ** value**
ALT (U/L)	1.004 (1.002-1.006)	<0.001	1.003 (0.990-1.015)	0.126
AST (U/L)	1.005 (1.003-1.008)	<0.001	1.001 (0.991-1.011)	0.836
TB (umol/L)	1.009 (1.002-1.016)	0.011	1.004 (0.989-1.019)	0.629
WBC (*10^^9^/L)	1.117 (1.078-1.158)	<0.001	1.149 (1.046-1.261)	0.004
Lymphocyte (*10^^9^/L)	0.066 (0.037-0.119)	<0.001	0.297 (0.139-0.637)	0.002
Creatinine (umol/L)	1.006 (1.002-1.011)	0.004	1.006 (1.001-1.010)	0.062
LDH (U/L)	1.007 (1.005-1.009)	<0.001	1.008 (1.004-1.011)	< 0.001
D-dimer (FEUmg/L)	1.074 (1.045-1.105)	<0.001	1.006 (0.958-1.057)	0.800
CRP (mg/L)	1.020 (1.016-1.024)	<0.001	1.007 (1.001-1.014)	0.020
PCT (ng/ml)	1.024 (1.007-1.041)	0.006	1.025 (1.008-1.042)	0.093
Cardiac troponin T (ng/ml)	2.793 (1.271-6.136)	0.011	2.363 (0.670-8.329)	0.181
NT-proBNP (per 100 pg/ml increase)	1.006 (1.005-1.008)	<0.001	1.002 (0.998-1.006)	0.436
Oxygen saturation on room air	0.888 (0.860-0.917)	<0.001	0.955 (0.922-0.990)	0.011

CI, confidence interval; OR, odds ration; ALT, alanine aminotransferase; AST, aspartate transaminase; TB, total bilirubin; WBC, white blood cell; eGFR, estimated glomerular filtration rate; LDH, lactate dehydrogenase; CRP, C-reactive protein; PCT, procalcitonin; NT-proBNP, N-terminal forebrain natriuretic peptide.

### Development of a practical scoring model for critical illness

In order to simplify the score and increase its reproducibility and applicability in other hospitals, the values of the five independent predictors mentioned above were transformed into categorical variables, and put into a multivariate logistic analysis including demographics, comorbidities and COVID-19 vaccination history. The result of multivariate analyses showed that history of malignancy was a significant predictor (OR: 6.084; 95% CI: 2.832-13.069; *p* < 0.001), and oxygen saturation < 90% on room air was a secondary positive predictor of critical illness (OR: 5.213; 95% CI: 2.839-9.573; *p* < 0.001). Other independent predictors for critical illness were chronic cardiac disease (OR: 3.288; 95% CI: 1.933-5.593; *p* < 0.001), CRP > 10 mg/L (OR: 3.259; 95% CI: 1.491-7.126; *p* = 0.003), chronic kidney disease (OR: 3.200; 95% CI: 1.863-5.498; *p* < 0.001), LDH > 250 U/L (OR: 3.113; 95% CI: 1.833-5.288; *p* < 0.001), COPD (OR: 3.040; 95% CI: 1.209-7.642; *p* = 0.018), age > 65 (OR: 2.917; 95% CI: 1.471-5.786; *p* = 0.002), non-vaccination (OR: 2.671; 95% CI: 1.356-5.259; *p* = 0.005), lymphocyte < 0.8*10^^9^/L (OR: 2.659; 95% CI: 1.570-4.504; *p* < 0.001), diabetes (OR: 2.451; 95% CI: 1.439-4.173; *p* = 0.001), cerebrovascular disease (OR: 2.036; 95% CI: 1.093-3.796; *p* = 0.025), and WBC > 10*10^^9^/L (OR: 1.933; 95% CI: 1.038-3.599; *p* = 0.038) (Table 3).

**TABLE 3:** Univariate and multivariate analysis of factors predicting critical illness.

Predictors	Univariate analysis		Multivariate analysis	
	**OR (95% CI)**	***p*** ** value**	**OR (95% CI)**	***p*** ** value**
Age > 65	6.603 (3.727-11.698)	<0.001	2.917 (1.471-5.786)	0.002
Male sex	1.895 (1.247-2.882)	0.003	1.013 (0.613-1.674)	0.742
WBC > 10 (*10^^9^/L)	6.032 (3.826-9.511)	<0.001	1.933 (1.038-3.599)	0.038
Lymphocyte < 0.8 (*10^^9^/L)	6.368 (4.180-9.703)	<0.001	2.659 (1.570-4.504)	<0.001
LDH > 250 (U/L)	8.925 (5.840-13.640)	<0.001	3.113 (1.833-5.288)	<0.001
CRP > 10 (mg/L)	12.816 (6.424-25.568)	<0.001	3.259 (1.491-7.126)	0.003
Oxygen saturation < 90% on room air	9.835 (6.175-15.665)	<0.001	5.213 (2.839-9.573)	<0.001
Hypertension	1.627 (1.076-2.458)	0.021	1.090 (0.536-1.978)	0.930
Diabetes	4.574 (3.034-6.895)	<0.001	2.451 (1.439-4.173)	0.001
Chronic cardiac disease	8.049 (5.302-12.219)	<0.001	3.288 (1.933-5.593)	<0.001
Chronic kidney disease	6.369 (4.193-9.673)	<0.001	3.200 (1.863-5.498)	<0.001
COPD	3.666 (1.763-7.622)	<0.001	3.040 (1.209-7.642)	0.018
Liver disease	2.585 (1.085-6.158)	0.032	1.844 (0.522-6.515)	0.342
Cerebrovascular disease	4.512 (2.838-7.174)	<0.001	2.036 (1.093-3.796)	0.025
Malignancy	3.470 (1.969-6.114)	<0.001	6.084 (2.832-13.069)	<0.001
Non-vaccination	2.837 (1.875-4.294)	<0.001	2.671 (1.356-5.259)	0.005

CI, confidence interval; OR, odds ration; WBC, white blood cell; LDH, lactate dehydrogenase; CRP, C-reactive protein; COPD, chronic obstructive pulmonary disease.

**FIGURE 2:**
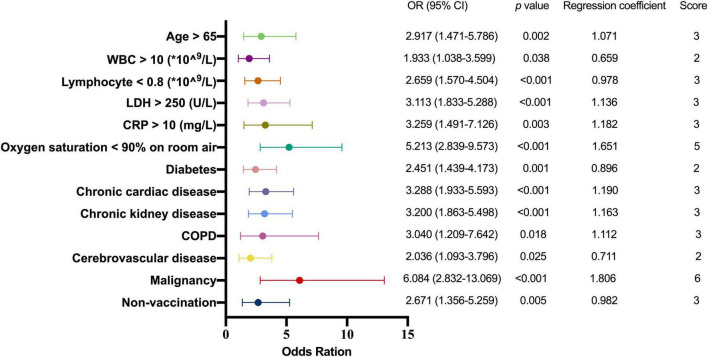
Multivariate logistic regression analysis of factors predicting critical illness. OR, odds ration; WBC, white blood cell; LDH, lactate dehydrogenase; CRP, C-reactive protein; COPD, chronic obstructive pulmonary disease.

**FIGURE 3:**
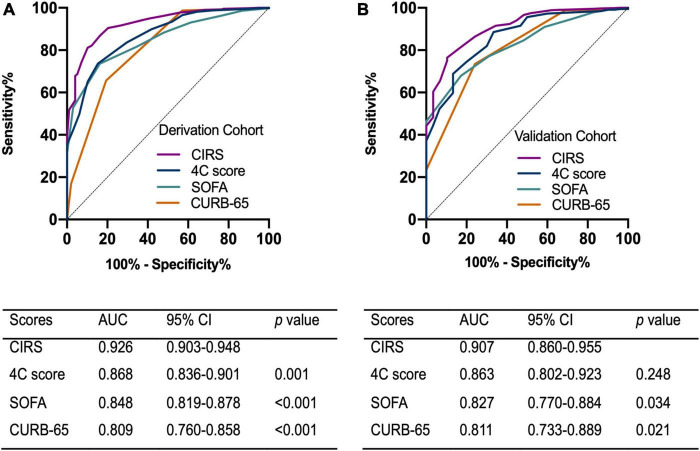
Receiver operating characteristic curve of the scoring models on the prediction of critical illness in **(A)** the derivation cohort **(B)** and the validation cohort. AUC, area under curve; CI, confidence interval; CIRS, critical illness risk score; SOFA, sequential organ failure assessment.

In order to develop a useful clinical predicting tool, relative weights were assigned according to the regression coefficient of each categorical variable (Figure 2), and each patient had a prognostic score of critical illness. The sum of scores from each of the thirteen predictors ranged from 0 to 36 in this study, and patients were divided into high-risk and low-risk groups with the cut-off value of 13. The area under the ROC (AUROC) of the derivation cohort was 0.926 (95% CI, 0.903–0.948), with sensitivity of 81.2% and specificity of 89.8% (Figure 3A).

### Validation of the clinical scoring model

The validation cohort included 738 patients with a median age of 66 years. Critical illness eventually developed in 30 of these patients and six died. The clinical and laboratory characteristics of the derivation and validation cohorts are listed in Supplementary Table 1. The two cohorts differed on CRP, history of hypertension, symptoms on admission including fever, cough and expectoration. Compared with derivation cohort, there were more patients with a history of hypertension (52.4% vs. 46.8%, *p* = 0.010), while fewer patients had fever (14.1% vs. 21.3%, *p* < 0.001), cough and expectoration (23.3% vs. 29.2%, p = 0.003) on admission in the validation cohort. The potency of the clinical scoring model to predict critical illness in patients with Omicron infection was assessed using the area under the ROC in the validation cohort. The AUROC of the validation cohort was 0.907 (95% CI, 0.860-0.955) (Figure 3B). The critical illness risk score (CIRS) when compared with the CURB-65, SOFA, and 4C mortality scores displayed an AUROC that was significantly higher in both cohorts. Furthermore, the better performance of the CIRS was further demonstrated by significant improvements in reclassification as assessed by continuous NRI and IDI in both cohorts (Supplementary Table 2).

**FIGURE 4:**
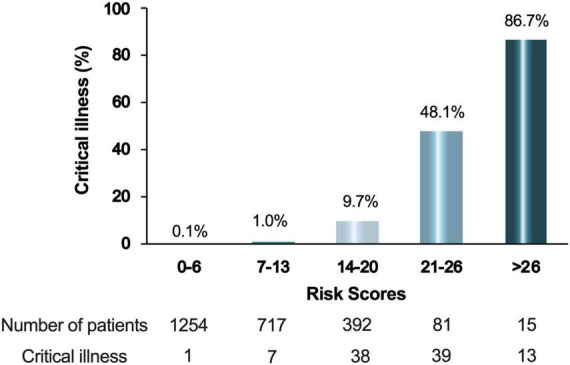
Percentage of critical ill patients according to the score. The integer scores were converted by rounding the odds ratios of the predictors and the final score was the sum of these values. Patients were divided into 5 groups according to their scores.

All 2459 patients in this study were pooled to further assess the correlation between the clinical scoring model and the rate of critical illness events. The score was categorized into two groups with high risk (score > 13) and low risk (score < 13) of critical illness. Among patients with score of 0-6, 0.1% had developed critical illness, as compared to 1.0% of patients with score of 7-13, 9.7% of patients with score of 14-20, 48.1% and 86.7% of patients with score of 21-26 and > 26, respectively (Figure 4). For the summed clinical scores of 0-13 and 14-36, the critical illness event was 0.1-1.0% and 9.7%-86.7%, respectively.

## Discussion

In this study, we tested the extent to which demographic, routine laboratory parameters, and comorbidities predicted the critical illness events. We found that age, LDH, CRP, WBC count, lymphocyte count, oxygen saturation, the history of malignancy, COPD, diabetes, chronic cardiac disease, chronic kidney disease, cerebrovascular disease, and non-vaccination were all independent predictors for critical illness. Furthermore, we developed and validated a clinical scoring model to predict the development of critical illness among hospitalized SARS-CoV-2 infected patients. The performance of this risk scoring model was satisfactory with accuracy based on AUROCs in both the derivation and validation cohorts.

COVID-19 is a heterogeneous disease of varying severity and prognosis (Samadizadeh et al., 2021). An increasing number of studies have analyzed the associated factors in patients with COVID-19. Previous studies have shown that the predictors of severe prognosis in patients with COVID-19 included age, sex, CRP, LDH, lymphocyte count, D-dimer, and comorbidity (including hypertension, diabetes, cardiovascular disease, respiratory disease) (Huang H. et al., 2020; Liang et al., 2020b; Wynants et al., 2020). These predictors coincide with most of those we have observed in the multivariable analysis and included in the predictive models. Some studies have shown that the increase of CRP in critical illness may be related to cytokine storms, which produce a series of inflammatory responses and cause disorders in peripheral WBCs (Chen et al., 2020), while the decrease in lymphocytes indicated that coronavirus consumed many immune cells and inhibited the body’s cellular immune function (Liu et al., 2017). LDH may be related to respiratory function and the increase of LDH will cause greater disease severity (Poggiali et al., 2020). In addition, some other studies have suggested that radiological findings, immunological index, tobacco and comorbidities as obesity may also be associated with a worse outcome (Huang Y. et al., 2020; Li et al., 2020; Ruch et al., 2020; Zheng et al., 2020; Surme et al., 2021). We could not assess the effect of the variables such as body mass index and smoking history since the data from electronic medical records were incomplete.

In addition, in our study, the finding was expected that age and complications were important predictors for critical illness. An analysis using propensity score matching (PSM) was further conducted to reduce the effects of age and comorbidities. Based on a caliper set as 0.02, a 1:1match was achieved using the nearest neighbor-matching algorithm. After age-, sex- and comorbidities-matching, 196 patients (98 patients each for critical illness and non-critical illness groups) were analyzed to identify independent predictors of critical illness. Demographics and clinical characteristics between the two groups were shown in Supplementary Table 3. The result showed that WBC > 10*10^^9^/L, CRP > 10 mg/L, LDH > 250 U/L, lymphocyte < 0.8*10^^9^/L, Oxygen saturation < 90%, and non-vaccination were still independent predictors for critical illness (Supplementary Table 4). Therefore, patients with abnormal WBC, lymphocyte, LDH, CRP, Oxygen saturation, and non-vaccination were at a higher risk of developing critical illness than the general population, under the same age, sex, and presence of comorbidities.

Although most patients have mild or moderate disease, Omicron infection can progress to severe disease and result in acute respiratory distress syndrome, multiorgan failure and death (Wu and McGoogan, 2020). Since early intervention was helpful to improve the prognosis (Garcia-Del-Barco et al., 2021; Qian et al., 2022), early stratifying of patients based on disease severity is vital. CURB-65 is a commonly applied severity score in community-acquired pneumonia management, and it has also been previously validated in different populations of COVID-19 positive patients (Nguyen et al., 2020; Artero et al., 2021; Elmoheen et al., 2021). The SOFA score has been proven to have a high predictive value for intensive care unit (ICU) mortality in severely ill patients (Vincent et al., 1996; Liu et al., 2020). The 4C mortality score is a well-established model that has been validated internally and externally to predict the outcome of COVID-19 (Knight et al., 2020). In our study, we compared the predictive ability of the proposed CIRS model with the SOFA, CURB-65 and 4C mortality scores, based on the AUROC. We found that the CIRS performed best. Moreover, the IDI and NRI indices also supported this conclusion.

Compared with the original and the Delta strains, the pathogenicity of Omicron greatly weakened, while it has become extremely infectious (He et al., 2021). Our risk scoring model can divide patients into high-risk and low-risk groups at admission. The stratified analysis of patients in the high-risk group found that patients with higher scores had higher critical illness rate. These findings indicated that when managing COVID-19 patients, special attention should be paid to those patients with higher risk score.

This study had several limitations. First, this is a single-center and retrospective study. Second, only 98 patients who have developed critical illness in our study, and it is likely that the AUROC overestimates the predictive power of the model. Third, although in our study, patients were divided into derivation cohort and validation cohort, the risk scoring model has a certain predictive value, the model was not been verified in the external validation cohort. Moreover, a large-scale multicenter prospective study is warranted to validate the risk model in the future.

In summary, we developed a clinical scoring model to estimate the risk of developing critical illness among patients with Omicron infection based on thirteen variables commonly measured on admission to the hospital. We believe that the results from this study will help clinicians stratify risk and provide opportunity for early intervention.

## Data availability statement

The raw data supporting the conclusions of this article will be made available by the authors, without undue reservation.

## Ethics statement

The studies involving human participants were reviewed and approved by the Ethics Committee of Huashan Hospital, Fudan University. Written informed consent from the participants’ legal guardian/next of kin was not required to participate in this study in accordance with the national legislation and the institutional requirements.

## Author contributions

YZ, JH, FS, YuG, YiG, HaZ, FL, ZX, SM, HuZ, ZG, JY, YoZ, LQ, KM, RM, and JZ contributed to study concept and design. YZ and JH accessed and verified all the data and did the statistical analysis. YZ drafted the manuscript. YZ, JH, FS, YuG, YiG, and HaZ conducted the collection of clinical data. FL, ZX, SM, HuZ, ZG, JY, YoZ, and LQ contributed to the data interpretation. KM and RM did critical revision of manuscript for important intellectual content. JZ supervised the whole project. All authors have read and approved the final version of the manuscript.
