# Supplementary material for: A practical scoring model to predict the occurrence of critical illness in hospitalized patients with SARS-CoV-2 omicron infection
Source: Front Microbiol. 2022 Dec 19;13:1031231. doi: 10.3389/fmicb.2022.1031231 (PMC9806124; doi:10.3389/fmicb.2022.1031231)
Supplement: Supplementary file 1 [file Data_Sheet_1.docx]

Supplementary Material

# Supplementary Figures and Tables

## Supplementary Tables

**Supplementary Table 1. Demographics and clinical characteristics of the patients in derivation and validation cohorts.**

|  | Derivation | Validation | *p* value |
| --- | --- | --- | --- |
| Total (n) | 1721 | 738 |  |
| Male | 829 (48.2%) | 364 (49.3%) | 0.600 |
| Age (years) | 64.0 (49.0-73.0) | 66.0 (53.0‑73.5) | 0.229 |
| **Laboratory findings on admission** | |  |  |
| ALT (U/L) | 16.0 (11.0-25.0) | 16.0 (11.0-24.0) | 0.478 |
| AST (U/L) | 20.0 (16.0-26.0) | 20.0 (16.0-27.0) | 0.323 |
| TB (umol/L) | 8.0 (5.6-11.4) | 7.9 (5.5-11.1) | 0.235 |
| WBC (*10^^9^/L) | 5.2 (3.9-6.8) | 5.1 (3.9-6.6) | 0.213 |
| Lymphocyte (*10^^9^/L) | 1.2 (0.8-1.7) | 1.2 (0.8-1.6) | 0.702 |
| Creatinine (umol/L) | 76.0 (61.0-103.0) | 79.0 (61.0‑108.5) | 0.284 |
| eGFR (mL/min) | 84.1 (57.8-104.1) | 81.7 (55.6-102.2) | 0.854 |
| LDH (U/L) | 195.0 (166.0-232.5) | 193.0 (166.0-233.8) | 0.145 |
| D-dimer (FEUmg/L) | 0.6 (0.3-1.1) | 0.6 (0.3-1.0) | 0.176 |
| CRP (mg/L) | 8.0 (5.0-26.1) | 8.2 (5.0-24.8) | 0.016 |
| PCT (ng/mL) | 0.1 (0.1-0.5) | 0.1 (0.1-0.5) | 0.650 |
| cTnT (ng/mL) | 0.010 (0.007-0.033) | 0.010 (0.007-0.034) | 0.089 |
| NT-proBNP (pg/mL) | 137.0 (47.5-877.5) | 133.0 (44.3-760.0) | 0.418 |
| **Comorbidities, N (%)** |  |  |  |
| Hypertension | 805 (46.8%) | 387 (52.4%) | 0.010 |
| Diabetes | 433 (25.2%) | 184 (24.9%) | 0.905 |
| Chronic cardiac disease | 276 (16.0%) | 111 (15.0%) | 0.534 |
| Cerebrovascular disease | 201 (11.7%) | 91 (12.3%) | 0.647 |
| Chronic kidney disease | 260 (15.1%) | 133 (18.0%) | 0.071 |
| COPD | 66 (3.8%) | 17 (2.3%) | 0.054 |
| Liver disease | 44 (2.6%) | 21 (2.8%) | 0.682 |
| Malignancy | 131 (7.6%) | 48 (6.5%) | 0.333 |
| **Symptoms, N (%)** |  |  |  |
| Fever | 367 (21.3%) | 104 (14.1%) | <0.001 |
| Cough and expectoration | 503 (29.2%) | 172 (23.3%) | 0.003 |
| Pharyngalgia | 54 (3.1%) | 16 (2.2%) | 0.185 |
| Feeble | 210 (12.2%) | 80 (10.8%) | 0.337 |
| Runny nose | 33 (1.9%) | 8 (1.1%) | 0.139 |
| **Vaccination status, N (%)** |  |  |  |
| Unvaccinated | 685 (39.8%) | 298 (40.4%) | 0.789 |
| Partially vaccinated | 53 (3.1%) | 19 (2.6%) | 0.496 |
| Fully vaccinated | 455 (26.4%) | 207 (28.0%) | 0.409 |
| Booster vaccination | 528 (30.7%) | 214 (29.0%) | 0.405 |
| **Oxygen support need, N (%)** | |  |  |
| Non-invasive ventilation | 333 (19.3%) | 165 (22.4%) | 0.089 |
| High flow oxygen | 35 (2.0%) | 20 (2.7%) | 0.299 |
| Mechanical ventilation | 28 (1.6%) | 18 (2.4%) | 0.173 |
| **Medications, N (%)** |  |  |  |
| Ritonavir/Darunavir | 620 (36.0%) | 294 (40.2%) | 0.073 |
| Heparin | 419 (24.3%) | 204 (27.6%) | 0.085 |
| Glucocorticoid | 100 (5.8%) | 54 (7.3%) | 0.158 |
| Vasoactive drugs | 38 (2.2%) | 14 (1.9%) | 0.623 |

Values are expressed as median (IQR) or number of patients (%). ALT, alanine aminotransferase; AST, aspartate transaminase; TB, total bilirubin; WBC, white blood cell; eGFR, estimated glomerular filtration rate; LDH, lactate dehydrogenase; CRP, C-reactive protein; PCT, procalcitonin; cTnT, Cardiac troponin T; NT-proBNP, N-terminal forebrain natriuretic peptide; COPD, chronic obstructive pulmonary disease.

**Supplementary Table 2: Incremental effect of CIRS model on the prediction of critical illness.**

| Predictive model | | cNRI (95%CI) | *p* value | IDI (95%CI) | *p* value |
| --- | --- | --- | --- | --- | --- |
| **Derivation cohort** | CIRS |  |  |  |  |
|  | 4C score | 0.571 (0.371-0.771) | 0.005 | 0.104 (0.052-0.156) | <0.001 |
|  | SOFA | 0.706 (0.508-0.903) | <0.001 | 0.095 (0.034-0.155) | 0.002 |
|  | CURB-65 | 0.825 (0.636-0.915) | <0.001 | 0.144 (0.091-0.198) | <0.001 |
| **Validation cohort** | CIRS |  |  |  |  |
|  | 4C score | 0.384 (0.015-0.753) | 0.041 | 0.115 (0.008-0.221) | 0.034 |
|  | SOFA | 0.590 (0.230-0.950) | <0.001 | 0.123 (0.023-0.223) | 0.001 |
|  | CURB-65 | 0.671 (0.327-0.942) | <0.001 | 0.126 (0.041-0.210) | 0.003 |

cNRI, Continuous net reclassification index; IDI, integrated discrimination improvement; CI: confidence interval; CIRS, critical illness risk score; SOFA, sequential organ failure assessment. We calculated the continuous NRI and IDI using the R package “PredictABEL”, in comparison to that of the CIRS.

**Supplementary Table 3. Demographics and clinical characteristics of patients after propensity score matching.**

|  | Non-critical illness | Critical illness | *p* value |
| --- | --- | --- | --- |
| Total (n) | 98 | 98 |  |
| Male | 60 (61.2%) | 63 (64.3%) | 0.658 |
| Age (years) | 77.0 (71.0-87.0) | 81.0 (70.3‑89.8) | 0.386 |
| **Laboratory findings on admission** | |  |  |
| ALT (U/L) | 15.0 (10.8-23.0) | 19.0 (11.0-34.5) | 0.023 |
| AST (U/L) | 20.0 (16.0-26.5) | 28.0 (18.3-49.5) | 0.031 |
| TB (umol/L) | 8.7 (5.9-12.6) | 9.9 (6.9-14.4) | 0.063 |
| WBC (*10^^9^/L) | 5.7 (4.4-7.2) | 7.4 (4.8-11.3) | <0.001 |
| Lymphocyte (*10^^9^/L) | 1.1 (0.8-1.5) | 0.7 (0.5-1.0) | <0.001 |
| Creatinine (umol/L) | 106.0 (72.0-309.5) | 128.0 (70.3‑450.5) | 0.335 |
| eGFR (mL/min) | 54.0 (26.5-82.0) | 40.5 (12.0-78.5) | 0.592 |
| LDH (U/L) | 228.0 (203.0-265.0) | 300.0 (219.8-429.8) | <0.001 |
| D-dimer (FEUmg/L) | 0.9 (0.5-2.0) | 2.4 (1.1-5.0) | <0.001 |
| CRP (mg/L) | 20.3 (5.1-53.2) | 72.1 (34.3-111.7) | <0.001 |
| PCT (ng/mL) | 0.2 (0.1-0.7) | 0.6 (0.2-2.7) | 0.004 |
| cTnT (ng/mL) | 0.030 (0.020-0.080) | 0.080 (0.040-0.183) | <0.001 |
| NT-proBNP (pg/mL) | 608.5 (215.0-2745.0) | 3090.0 (1339.0-21822.0) | <0.001 |
| **Comorbidities, N (%)** |  |  |  |
| Hypertension | 65 (66.3%) | 63 (64.3%) | 0.764 |
| Diabetes | 49 (50.0%) | 48 (49.0%) | 0.886 |
| Chronic cardiac disease | 40 (40.8%) | 41 (41.8%) | 0.885 |
| Cerebrovascular disease | 24 (24.5%) | 27 (27.6%) | 0.625 |
| Chronic kidney disease | 35 (35.7%) | 39 (39.8%) | 0.601 |
| COPD | 13 (13.3%) | 9 (9.2%) | 0.365 |
| Liver disease | 6 (6.1%) | 7 (7.1%) | 0.774 |
| Malignancy | 12 (12.2%) | 16 (16.3%) | 0.414 |
| **Symptoms, N (%)** |  |  |  |
| Fever | 15 (15.3%) | 33 (33.7%) | 0.003 |
| Cough and expectoration | 32 (32.7%) | 47 (48.0%) | 0.029 |
| Pharyngalgia | 5 (5.1%) | 8 (8.2%) | 0.389 |
| Feeble | 14 (14.3%) | 20 (20.4%) | 0.258 |
| Runny nose | 3 (3.1%) | 5 (5.1%) | 0.721 |
| **Vaccination status, N (%)** |  |  |  |
| Unvaccinated | 57 (58.2%) | 81 (82.7%) | 0.020 |
| Partially vaccinated | 4 (4.1%) | 1 (1.0%) | 0.369 |
| Fully vaccinated | 14 (14.3%) | 4 (4.1%) | 0.024 |
| Booster vaccination | 23 (23.5%) | 12 (12.2%) | 0.040 |
| **Oxygen support need, N (%)** | |  |  |
| Non-invasive ventilation | 25 (25.5%) | 36 (36.7%) | 0.090 |
| High flow oxygen | 5 (5.1%) | 22 (22.4%) | <0.001 |
| Mechanical ventilation | 0 (0%) | 39 (39.8%) | <0.001 |
| **Medications, N (%)** |  |  |  |
| Ritonavir/Darunavir | 38 (38.8%) | 42 (42.9%) | 0.561 |
| Heparin | 32 (32.7%) | 55 (56.1%) | <0.001 |
| Glucocorticoid | 7 (7.1%) | 36 (36.7%) | <0.001 |
| Vasoactive drugs | 0 (0%) | 52 (53.1%) | <0.001 |

Values are expressed as median (IQR) or number of patients (%). ALT, alanine aminotransferase; AST, aspartate transaminase; TB, total bilirubin; WBC, white blood cell; eGFR, estimated glomerular filtration rate; LDH, lactate dehydrogenase; CRP, C-reactive protein; PCT, procalcitonin; cTnT, Cardiac troponin T; NT-proBNP, N-terminal forebrain natriuretic peptide; COPD, chronic obstructive pulmonary disease.

**Supplementary Table 4.** Univariate and multivariate analysis of factors predicting critical illness in matched patient sub-groups.

| Predictors | Univariate analysis | |  | Multivariate analysis | |
| --- | --- | --- | --- | --- | --- |
|  | OR (95% CI) | *p* value |  | OR (95% CI) | *p* value |
| WBC > 10 (*10^^9^/L) | 4.575 (2.042-10.254) | <0.001 |  | 2.567 (1.043-6.317) | 0.040 |
| Lymphocyte < 0.8 (*10^^9^/L) | 2.718 (1.526-4.843) | 0.001 |  | 2.388 (1.187-4.804) | 0.015 |
| LDH > 250 (U/L) | 1.864 (1.055-3.293) | 0.032 |  | 2.156 (1.056-4.403) | 0.035 |
| CRP > 10 (mg/L) | 4.575 (2.042-10.254) | <0.001 |  | 3.217 (1.213-8.533) | 0.019 |
| Oxygen saturation < 90% on room air | 6.303 (2.622-15.151) | <0.001 |  | 5.835 (2.164-15.736) | <0.001 |
| Non-vaccination | 3.476 (1.918-6.297) | <0.001 |  | 4.401 (2.121-9.131) | <0.001 |

CI, confidence interval; OR, odds ration; WBC, white blood cell; LDH, lactate dehydrogenase; CRP, C-reactive protein.

## Supplementary Figures


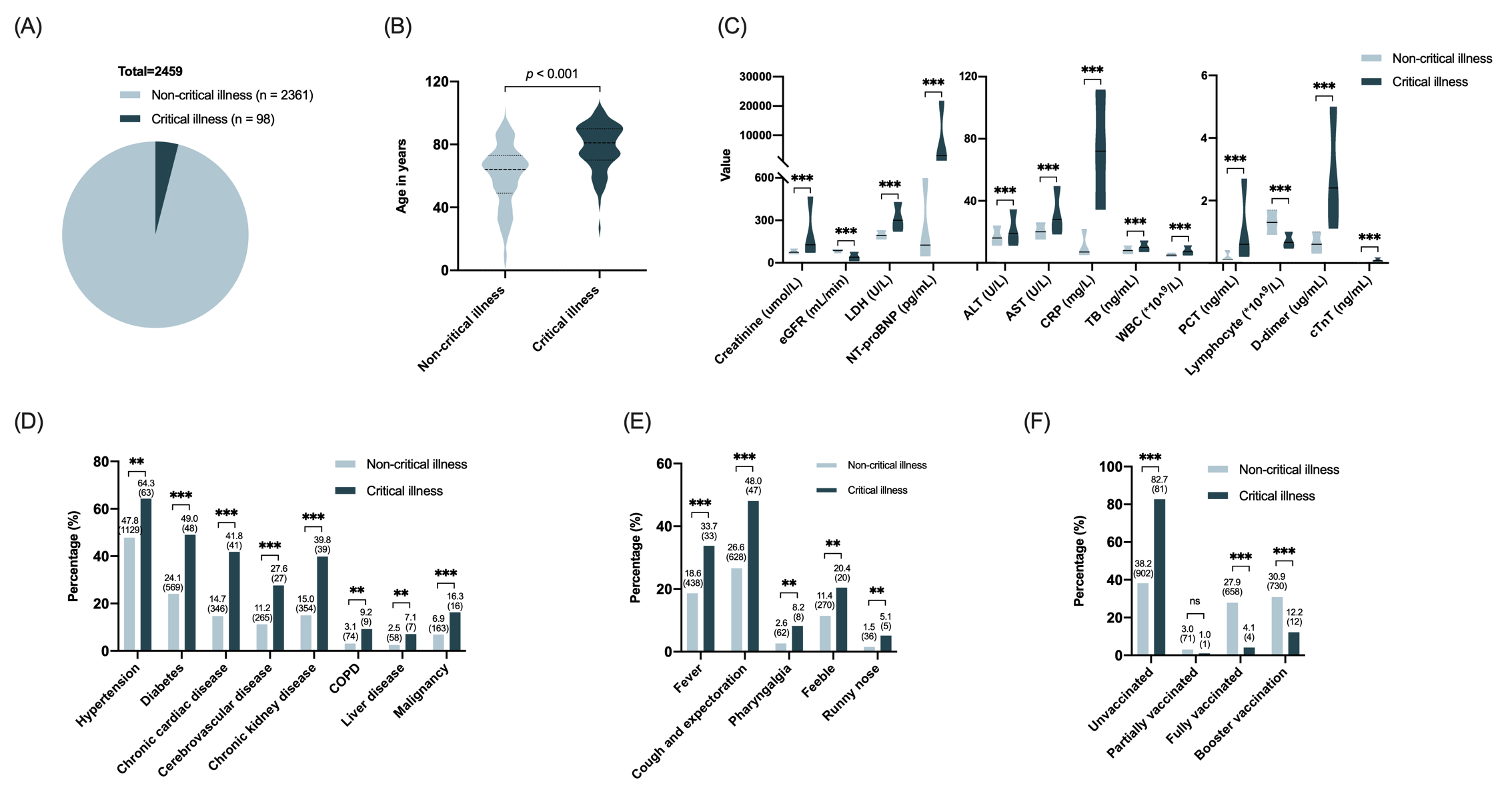


**Supplementary Figure 1.** Characteristics of patients in the study. (A) A total of 98 patients eventually developed critical illness. (B) Age, (C) laboratory examination, (D) comorbidities, (E) clinical symptom, (F) and vaccination status on admission in all critical ill and non-critical ill patients. ns, no significance. *p < 0.05, **p < 0.01, ***p < 0.001.
